# A sentiment analysis on online psychiatrist reviews to identify clinical attributes of psychiatrists that shape the therapeutic alliance

**DOI:** 10.3389/fpsyt.2023.1174154

**Published:** 2023-06-15

**Authors:** Soo Hwan Park, Christopher P. Cheng, Nicholas J. Buehler, Timothy Sanford, William Torrey

**Affiliations:** ^1^Geisel School of Medicine at Dartmouth, Hanover, NH, United States; ^2^Department of Psychiatry, Dartmouth Health, Lebanon, NH, United States; ^3^Icahn School of Medicine at Mount Sinai, New York, NY, United States; ^4^Northwestern University Feinberg School of Medicine, Chicago, IL, United States

**Keywords:** physician rating websites, sentiment analysis, psychiatrist, therapeutic alliance, medication side effects

## Abstract

**Background:**

While online reviews from physician rating websites are increasingly utilized by healthcare providers to better understand patient needs, it remains difficult to objectively identify areas for improvement in providing psychiatric care.

**Objectives:**

To quantitatively characterize the sentiment of online written reviews of psychiatrists to determine clinical attributes that can be strengthened to improve psychiatrists’ therapeutic alliance with their patients.

**Materials and methods:**

Sentiment scores of 6,400 written reviews of 400 US-based psychiatrists on a US-based online physician rating website were obtained through a natural-language-processing-based sentiment analysis. Relationships among sentiment scores, average star ratings, and demographics were examined. Linguistic analyses determined words and bigrams that were highly associated with reviews with the most positive and negative sentiment.

**Findings:**

Sentiment scores were significantly correlated with average star ratings of the psychiatrists (*R* = 0.737, *p* < 0.001). Psychiatrists who were younger (< 56 years old) and/or practiced in the Northeast had significantly higher average star ratings than those older and/or practicing in the Southwest. Frequency analysis showed that positive reviews most frequently contained “time” (*N* = 1,138) and “caring” (*N* = 784) while negative reviews most frequently contained “medication” (*N* = 495) and “time” (*N* = 379). Logistic regression analysis revealed that reviews were more likely to be considered positive when they included “great listener” (OR = 16.89) and “comfortable” (OR = 10.72) and more likely to be negative when they included “meds” (OR = 0.55) and “side effect” (OR = 0.59).

**Conclusion:**

Psychiatrists who are younger and located in the Northeast receive more positive reviews; there may be potential for demographic bias among patient reviewers. Patients positively rate psychiatrists who make them feel heard and comfortable but negatively rate encounters centered around medications and their side effects. Our study lends quantitative evidence to support the importance of thorough and empathetic communication of psychiatrists in building a strong therapeutic alliance.

## 1. Introduction

The therapeutic alliance, a patient’s interpersonal relationship with the patient’s psychiatrist, is a critical component of psychiatric care that significantly impacts outcomes, yet it remains difficult to quantitatively characterize and identify areas for improvement (1–3). Physician rating websites (PRWs) summarize patients’ complex experiences in the form of reviews and ratings and may serve as a window into the strength of the therapeutic alliance psychiatrists form with their patients (4–6). Although PRWs have been quantitatively analyzed in other medical specialties to describe physicians’ characteristics that affect care (7–9), no studies to date have leveraged PRWs to determine clinical attributes of psychiatrists that may affect their therapeutic alliance with their patients.

Sentiment analysis is a machine learning-based technique for rating positive or negative use of language in text that recently led to an increased understanding of clinical contributors to patient satisfaction in surgical fields (10, 11). In the field of psychiatry, previous studies have demonstrated the validity of sentiment analysis among antidepressant users and for online mental health consultation services (12, 13). Specifically, while the general attitude toward online mental health consultation services and the usefulness of online reviews in China have previously been examined, no studies to date have elucidated impactful attributes of psychiatrists that can improve patient experiences during their mental health visits in the United States using PRWs (13). To that end, the present study aimed to utilize sentiment analysis on online written reviews of psychiatrists from PRWs to identify demographic and clinical characteristics strongly associated with reviews with the most positive and negative sentiment.

## 2. Materials and methods

### 2.1. Data acquisition

Publicly available star-rating reviews (ratings out of five stars) as well as online written reviews of psychiatrists were gathered from healthgrades.com. This website was selected as it was one of the top websites suggested by an online search engine and without a firewall restricting automated scraping techniques used to collect reviews and demographic data (age, gender, geographic region) pertaining to the psychiatrists. Scraping was accomplished by parsing the HTML code of Healthgrades physician pages. Psychiatrists with fewer than seven reviews (10) were excluded to accurately calculate average star ratings and sentiment scores, as this was determined to be the optimal threshold for a strong association between sentiment scores and star ratings.

### 2.2. Sentiment analysis and VADER score calculation

In order to quantify the sentiment of each online written review, the Valence Aware Dictionary and sEntiment Reasoner (VADER) sentiment analysis package was employed in Python 3.7. The rule-based VADER package is part of a larger natural language processing library, the Natural Language Toolkit (NLTK) python library. For its comprehensive ability to analyze the sentiment of sentences, VADER has seen increased application in healthcare, particularly in analysis of social media data and reviews of surgeons (10, 14).

In short, the VADER package is able to take in written prose and assign a sentiment score of the sentence. The package performs its calculation mainly by utilizing a word lexicon developed by ten independent human raters, who scored 80% or higher on a standardized college-level reading comprehension test and were trained for online sentiment rating to ensure inter-rater consistency (15). For each word in the dictionary, the human raters attributed scores ranging from −4 (“Extremely Negative”) to +4 (“Extremely Positive”), with 0 reflecting a neutral sentiment. By scanning the inputted sentences for words in its lexicon, VADER generates a summed and normalized score between −1 and +1. In addition, it is able to consider the effects of emphasizing features of a sentence like capitalization and punctuation, as well as the impact of negators (i.e., “not”) and modifiers (i.e., “extremely”) on the sentiment of the lexicon-drawn words. For example, “extremely compassionate” would impact the overall sentiment more positively, and “not compassionate” would contribute more negatively.

### 2.3. Model validation

We performed a linear regression analysis to confirm the relationship between the average sentiment analysis score for each provider with their average star score, which supported the use of our inclusion and exclusion criteria for subsequent analyses.

### 2.4. Data analysis

In order to examine how different demographic features may impact average star ratings and sentiment scores of written reviews, we performed Student’s *t*-tests for age and sex analyses. The ages of the psychiatrists were used to divide them into cohorts of individuals above and below 56 years old, as 56 years split the distribution of psychiatrists the most evenly. Because some psychiatrists did not have their age listed on Healthgrades, these psychiatrists were not included in the age analysis. Physicians were also grouped by region of practice into five categories: Northeast, Southeast, Southwest, Midwest, and West following definitions set by National Geographic.^1^ One-way ANOVA with subsequent pairwise Tukey tests were conducted to examine the relationship between location and physician ratings and sentiment scores.

Subsequently, a word frequency analysis was conducted to determine the most prevalent words and word pairs (bigrams) used to write the most positive and negative reviews. We independently assessed the best reviews that had a sentiment score of greater than 0.5 as well as the worst reviews with a negative overall sentiment score in order to determine the most common content in each. To further confirm how frequently found clinically relevant words/bigrams impact the overall sentiment of the review, we performed a multivariate logistic regression on these clinically relevant words and bigrams to examine the impact of their association with a positive or negative sentiment score.

## 3. Results

### 3.1. Psychiatrist demographics

The application of the inclusion criteria described previously resulted in the initial collection of 8,206 reviews distributed across 2,066 psychiatrists; after removing 1,666 psychiatrists through the exclusion criteria, 400 psychiatrists and 6,400 online reviews were ultimately included for analysis. All demographic information was also gathered from healthgrades.com. The providers’ age, sex identity, and location of practice were scraped along with the associated reviews from the HTML code and have been summarized in Table 1.

**TABLE 1:** Demographic characteristics of the analyzed 400 psychiatrists pulled from healthgrades.com.

Demographics	*N*	%
**Gender**		
Male	227	56.8
Female	173	43.2
**Age^a^**		
< 56 years/old	171	49.6
≥ 56 years/old	174	50.4
**Location**		
Northeast	79	19.8
Southeast	112	28
Southwest	91	22.8
Midwest	62	15.5
West	56	14

^a^Some psychiatrists did not have their age listed and hence were not included in the age analysis.

### 3.2. Model validation: linear regression

**FIGURE 1:**
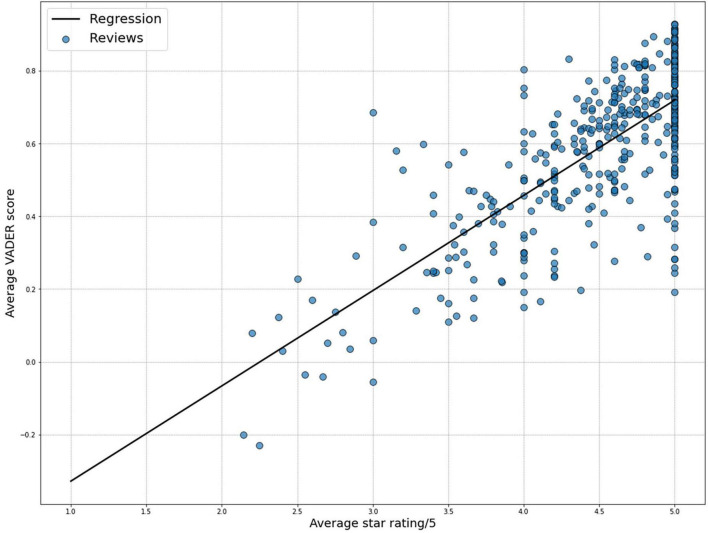
Significant correlation (*R* = 0.737, *p* < 0.001) between the psychiatrists’ average star ratings and the average sentiment scores generated using the Valence Aware Dictionary and sEntiment Reasoner (VADER) package.

The linear regression model examining the relationship between average star ratings and average sentiment analysis scores demonstrated a significant correlation (Figure 1, *R* = 0.737; *p* < 0.001). The statistically significant correlation validated the utility of sentiment analysis scores generated by VADER for linguistic analyses.

### 3.3. Model validation and demographic analyses

There was a significant difference in star rating between older and younger psychiatrists (*t* = 2.060; *p* < 0.05), with younger psychiatrists having a larger average star rating (< 56: mean = 4.513) than older psychiatrists (≥ 56: mean = 4.376). No statistically significant difference in sentiment analysis score was found between the two age groups. Gender analysis revealed no statistically significant difference in average star ratings or sentiment analysis scores (*p* > 0.05) between male and female psychiatrists. Location analysis showed an overall significant difference in star rating (*F* = 2.479; *p* = 0.044), but not in sentiment analysis score. Subsequent Tukey’s pairwise comparisons revealed a significant difference in average star rating score between the psychiatrists in the Northeast (mean = 4.588) and the Southwest (mean = 4.320) regions of the United States (*p*-FWE = 0.035). The results of the analyses have been summarized in Table 2.

**TABLE 2:** Bias analysis comparing star scores and sentiment scores with sex, age, and region.

Variable	Age				Gender				Region					
	< 56	≥ 56	*T*	*P*-value	Male	Female	*T*	*P*-value	NE	SE	SW	MW	W	*P*-value
Star reviews	4.513	4.376	2.060	0.040*	4.432	4.494	1.906	0.057	4.589	4.454	4.32	4.418	4.552	0.044*
Sentiment scores	0.589	0.559	1.303	0.194	0.592	0.59	1.799	0.857	0.624	0.554	0.548	0.563	0.622	0.056

*p < 0.05. NE, northeast; SE, southeast; SW, southwest; MW, midwest; W, west.

### 3.4. Linguistic analyses

Single-words that were most frequently used in the most positive and negative online written reviews were identified. Only words that were clinically relevant and meaningful were included. For example, words like “great” and “recommend” were frequently used but did not add to the clinical understanding of why the provider was “great,” and were hence omitted. The identified high-frequency words from both positive and negative reviews mainly focused on the length of the visit, the demeanor of providers and staff, and medication. The most positive reviews included “time,” “caring,” “care,” “staff,” “treatment,” “kind,” and “listens.” The most negative reviews included “time,” “depression,” “medication,” “staff,” “meds,” “treatment,” “care,” “medications,” and “anxiety.”

Frequency analysis also revealed the most frequently used clinically relevant bigrams from the most positive and negative reviews. The top reviews focused on the behavioral attributes of the providers: “takes, time,” “easy, talk,” “cares, patients,” “office, staff,” “truly, cares,” “feel, comfortable,” and “time, listen.” The worst reviews included bigrams relevant to medications and time during and prior to the visit: “depression, anxiety,” “side, effects,” “takes, time,” “waiting, room,” “front, desk,” “appointment, time,” and “staff, rude.” These terms and their frequencies have been summarized in Table 3.

**TABLE 3:** Clinically relevant single-word and bigram frequency analysis of reviews with the most positive and negative sentiment.

Positive sentiment reviews				Negative sentiment reviews			
Word	Frequency	Bigram	Frequency	Word	Frequency	Bigram	Frequency
Time	1,138	Takes time	234	Meds/medication(s)	495	Depression anxiety	34
Caring	784	Easy talk	215	Time	379	Side effects	30
Care	784	Cares patients	200	Depression	217	Takes time	25
Staff	679	Office staff	152	Staff	187	Waiting room	19
Treatment	639	Truly cares	128	Treatment	149	Front desk	18
Kind	587	Feel comfortable	116	Care	146	Appointment time	16
Listens	561	Time listen	97	Anxiety	129	Staff rude	15

A multivariate logistic regression was performed to determine how much certain clinically relevant keywords contribute to the review being a positive or a negative review. Words and bigrams strongly associated with positive reviews included “listens,” “great listener,” “takes time,” “kind,” “empathetic,” “comfortable,” “caring,” and “compassionate.” Words and bigrams associated with negative reviews included “meds,” “side effect,” “complication,” and “waiting room.” The odds ratios of these words, which reflect the odds of the terms receiving an overall positive or negative sentiment score, have been summarized in Table 4.

**TABLE 4:** Multivariate logistic regression of clinically relevant words and bigrams.

Key word	2.5% CI	97.5% CI	OR	*P*-value
Takes time	2.00	4.75	3.09	< 0.01**
Listens	2.88	4.98	3.79	< 0.01**
Great listener	4.06	70.29	16.89	< 0.01**
Kind	4.57	8.74	6.32	< 0.01**
Waiting room	0.08	0.55	0.21	< 0.01**
Meds	0.44	0.69	0.55	< 0.01**
Side effect	0.35	0.99	0.59	0.049*
Complication	0.01	0.99	0.10	0.049*
Empathetic	2.16	9.82	4.61	< 0.01**
Comfortable	5.81	19.78	10.72	< 0.01**
Caring	6.47	12.69	9.06	< 0.01**
Compassionate	3.38	6.84	4.81	< 0.01**

**p < 0.01, *p < 0.05.

## 4. Discussion

Psychiatrists have recognized the need to leverage objective feedback to understand what patients expect from their mental health services and deliver the best care (16). While several patient satisfaction scales of psychiatrists have been developed and utilized to understand patient needs (17, 18), PRWs offer a more naturalistic and easily accessible set of reviews that psychiatrists can use to improve their services. The present study examines how patients are reviewing their psychiatrists online in the US by identifying words that are strongly associated with positive and negative reviews, in addition to studying the relationships between demographic factors, star ratings, and sentiment scores. Demographic analyses revealed that younger psychiatrists have significantly higher star ratings than older psychiatrists. Psychiatrists who practice in the Northeast region of the US also received significantly higher star ratings than psychiatrists who practice in the Southwest region. Linguistic analyses showed that positive reviews tend to have a high concentration of single words and bigrams relating to the amount of time taken by the psychiatrists to listen to their patients as well as words pertaining to the care and empathy that the psychiatrists showed for their patients. While negative reviews also emphasized the amount of time that psychiatrists took for their patients, they tended to focus more on medications and their side effects. To our knowledge, this is the first study that explores how patients are reviewing their psychiatrists on PRWs using a sentiment analysis approach.

Our finding that younger psychiatrists received higher star ratings than older psychiatrists is consistent with previous studies on physician reviews in different specialties. Several other studies have shown that younger physicians tend to have higher ratings than older physicians (19, 20). Considering that there was no significant difference in sentiment scores between the two groups, it is possible that older psychiatrists have been providing care for longer and thus had more time to accumulate negative ratings (21). It is also possible that older psychiatrists handle more difficult cases than younger psychiatrists, increasing the likelihood of their obtaining lower star ratings. Because different psychiatric subspecialties have differing patient demographics, more targeted analyses focusing on psychiatrists in specific subspecialties may reveal bias present in such populations and its overall effects on the sentiment of written reviews and star ratings.

Further demographic analyses showed that psychiatrists who practice in the Northeast region of the US received higher star ratings than those in the Southwest region. This finding may be tied to differential proportions of substance use and mental health facilities that only accept certain types of insurance. Previous studies have shown that the proportion of substance use facilities accepting Medicaid is lower in many Southern states than in other areas of the United States (22). Alternatively, other studies have demonstrated that general treatment prevalence in the South and Midwest was lower, relative to the Northeast (23). It is important to note that different policies and ways of practice contribute to the structural forces that ultimately shape longitudinal care and patient satisfaction (24).

Our linguistic analyses demonstrated that the reviews of psychiatrists with the most positive sentiment included words and bigrams pertaining to “time,” “listening,” and “caring.” These findings lend more quantitative and objective support to the existing evidence that highlights how much a psychiatrist’s particular interpersonal skills matter in shaping patient satisfaction (17). For example, the concentration on words related to “listen” and “care” aligns with some of the major categories defined in a recent patient-derived patient satisfaction scale of psychiatrists (17). Indeed, previous studies have shown that star rating is primarily driven by physicians’ bedside manner (25). Along this line of work, our findings emphasize certain behavioral characteristics that may guide psychiatrists in strengthening their therapeutic alliance with their patients.

On the other end of the spectrum, our linguistic analyses showed that words related to “time,” “medications,” and “side effects” significantly contribute to reviews having a higher degree of negative sentiment. It has been shown that most negative reviews online focus on patients’ interaction with psychiatrists and clinic staff (26). Specifically, as “time” seems to be critical in shaping patients’ perception of psychiatrists, if psychiatrists can make their patients feel as though they are taking their time for the patients, patients may view and rate their psychiatrists more favorably. This is much easier to do, of course, if the flow of daily work actually includes adequate time to thoughtfully engage in care (27). Additionally, previous studies have demonstrated that denials of requests for medication were associated with significantly lower patient satisfaction (28). Furthermore, physicians’ communication ability that provides clear medication consumption behavior also tended to have higher returns (5). Consistent with these findings, our results further highlight the significance and impact of medication selection, education, shared decision making, and their adverse effects on how favorably patients perceive their psychiatrists (29).

There are several limitations to our study. First, the 400 psychiatrists whose reviews were analyzed in this study represent a small subsection of the 38,411 actively practicing psychiatrists in the United States (30). Hence, patient reviews of these psychiatrists may not represent the full spectrum of patient perceptions of different forms of psychiatric care, either in the United States or abroad. Nevertheless, the 6,400 patient reviews examined in this sample nevertheless are on par with the sample size found in other sentiment analysis studies of patient reviews in mental healthcare (12). Given today’s accessibility of different publicly available physician rating websites across the globe, future studies may extend this approach by including other physician rating platforms and identifying common attributes of psychiatrists that positively shape patients’ perception of psychiatrists. Second, we did not consider the demographic distribution of patients who submit ratings and scores on PRWs. Patients’ characteristics were unavailable on the publicly accessible website utilized in this study and therefore not controlled. It is likely that younger patients who are more technologically literate compared to their older counterparts leave more online reviews; if the younger patients demonstrate a bias toward younger psychiatrists, the distribution of patients who leave reviews may have differentially affected the ratings of their psychiatrists. Thirdly, it is possible that single reviewers may have submitted multiple reviews for the same provider. Even if the phrases used in the written reviews by the single reviewers are unique, the overall sentiment and the star ratings may have accumulated and emphasized psychiatrists skewed toward one particular preference. Finally, the study did not consider the heterogeneity of psychiatric subspecialties. Though we were concerned with the general trend of the reviews and ratings of psychiatrists on PRWs, further analyses looking at specific subspecialties of psychiatrists may reveal trends and biases within patient-provider interactions specific to that patient population as well as their effects on the overall sentiment and ratings of psychiatrists on PRWs.

In conclusion, the present study conducted a sentiment analysis of reviews of psychiatrists from PRWs to determine demographic biases in the reviews and highlight attributes of psychiatrists that are associated with either positive or negative sentiment. By generating objective sentiment scores of the written reviews and identifying behavioral and clinical characteristics that patients care the most about, psychiatrists can gain greater insight on how to ameliorate their care to reinforce their therapeutic alliance with their patients. Further analyses examining how patients rate their psychiatrists are encouraged to include similar analyses on other PRWs to evaluate whether or not the overall online behavior of patients is consistent with our findings from healthgrades.com.

## Data availability statement

The original contributions presented in this study are included in the article/supplementary material, further inquiries can be directed to the corresponding author.

## Author contributions

SP contributed to the analysis and interpretation of the results and to the composition of the manuscript. CC contributed to the design of the analysis plan. NB, TS, and WT supported the interpretation and analysis of the data. WT contributed to the study design and edited the manuscript. All authors have critically reviewed the manuscript and approved the final version of the manuscript.

## Footnotes

1
https://education.nationalgeographic.org/resource/united-states-regions
